# Quantifying the association between rural older adult daily internet use duration and affective disorders: an empirical study based on propensity score matching

**DOI:** 10.3389/fpubh.2025.1722829

**Published:** 2026-01-13

**Authors:** Yudong Miao, Yixi Wang, Zhiping Guo, Tao Li, Jingming Wei, Zhanlei Shen, Dongfang Zhu, Jingbao Zhang, Jiajia Zhang, Mingyue Zhen, Xinran Li, Jinxin Cui, Clifford Silver Tarimo, Qingyong Lu, Jiaxin Han, Lingxiao Mou, Jingwei Qin

**Affiliations:** 1 Department of Health Management, College of Public Health, Zhengzhou University, Zhengzhou, Henan, China; 2 Henan Key Laboratory of Chronic Disease Management, Fuwai Central China Cardiovascular Hospital, Central China Fuwai Hospital of Zhengzhou University, Zhengzhou, Henan, China; 3 Institute of Mental Health, Peking University Sixth Hospital, Beijing, China; 4 Department of Science and Laboratory Technology, Dar es salaam Institute of Technology, Dar es Salaam, Tanzania

**Keywords:** affective disorders, empirical study, internet use duration, propensity score matching, rural older adult

## Abstract

**Background:**

There are significant disparities in internet usage among rural older adults, while affective disorder is an increasing public health concern. The potential link between these phenomena remains underexplored. This study investigates the association between internet use and affective disorder among rural older adults, to inform public health policies promoting appropriate internet engagement and psychological well-being.

**Methods:**

The baseline survey of the Northern China Lifestyle Medicine Cohort was conducted among rural older adult (≥65 years old) in China from July 2023 to January 2024. Questionnaires were used to collect data on the average daily usage schedule of the Internet and emotional disorders. Multivariate Logistic regression models were used to determine the factors influencing the prevalence of affective disorders. Propensity score matching (PSM) analysis was adopted to explore the relationship between Internet usage and emotional disorders.

**Results:**

A total of 9,924 participants were included in this study (43.0% were male), significantly higher than that of medium-term users (29.79%) and long-term users (25.29%). The prevalence of affective disorders is 18.54%, and there is a significant gender difference (males: 14.24%; females: 21.77%). After adjusting for potential confounders, we found that female gender was associated with a higher risk of affective disorders in both the medium-long internet usage group [aOR = 1.44, 95%CI (1.21–1.72)] and the short usage group [aOR = 1.39, 95%CI (1.15–1.68)], compared to males. For participants in the medium-long internet usage group, healthy sleep duration was associated with a lower risk of affective disorders [aOR = 0.68, 95%CI (0.58–0.79)]. The short prevalence of affective disorders among short-term Internet users is associated with healthy physical activity [aOR = 0.57, 95%CI (0.48–0.68)]. PSM analysis shows that after matching, the prevalence of affective disorders among short-term Internet users was higher than that among medium to long-term Internet users by 2.64%, and among all educational levels, the prevalence rate among women is higher than that among men.

**Conclusion:**

This study indicates that Internet use is associated with a lower prevalence of affective disorders among rural older adult. These findings emphasize the importance of formulating a public health strategy that integrates digital inclusion and mental health promotion.

**Clinical trial registration:**

https://www.chictr.org.cn/showprojEN.html?proj=206128.

## 1 Introduction

In recent years, thanks to the accelerated extension of digital infrastructure to rural areas, the continuous decline of tariff costs, and the progress of digital skills of vulnerable groups such as the older adult and low-income groups, Internet use in rural areas around the world has been rapidly popularized and is considered an important means to bridge the gap between urban and rural economic and social development. Meanwhile, affective disorder is increasingly recognized as a major public health concern (1). However, the potential link between these two phenomena remains insufficiently explored. This study aims to investigate the association between internet use and affective disorder among rural older adults, with the aim of informing public health policies that promote appropriate internet engagement and improve psychological well-being in this demographic (2).

This research imperative is underscored by recent national burden of disease data from China. Analyses of the Global Burden of Disease Study reveal a persistent and increasing burden of depressive disorders among middle-aged and older adults (aged 45–89) over the past three decades, highlighting mental health as a critical challenge in the context of rapid population aging and social transition (3). In this landscape, the digital inclusion of older adults, particularly those in rural areas who may face compounded vulnerabilities due to geographic isolation and fewer healthcare resources, emerges as a potential socio-behavioral determinant of mental health that warrants urgent empirical investigation.

Furthermore, affective disorders are highly prevalent among rural older adults. Relevant epidemiological surveys indicate that a significant proportion of older adults in China experience varying degrees of mental health issues, with prevalence rates increasing with age. These concerns are particularly common among those with chronic diseases, those who have lost their only child, or those lacking adequate social support (4). A previous meta-analysis reported a global prevalence of depressive symptoms among older adults as long as 35.1% (5). Affective disorders are associated with various negative physical health outcomes, including sleep problems and comorbidities. If left unaddressed, they may progress to more severe consequences (6). In recent years, the internet has garnered increasing attention as a potential tool for psychological support and health promotion. The digital divide, however, hinders older adults’ ability to connect with others and reduces their avenues for socialization. Limited internet participation hinders older adults from fully accessing the benefits of modern digital services, including medical information, virtual social interactions, social media connections, and e-commerce opportunities (7). This situation may contribute to feelings of isolation and helplessness (8), serving as a risk factor for Affective disorders such as depression (9). Moderate internet use may potentially enhance social connections, facilitate access to health information, and provide emotional support for older adults, thereby potentially alleviating symptoms of anxiety and depression. However, excessive use or exposure to harmful content online can also have negative effects. While existing research has demonstrated a correlation between internet use and the mental health status of adolescents or urban-dwelling older adults (10, 11), evidence that specifically focuses on the rural older adult population remains scarce.

Meanwhile, existing research is gradually revealing a possible bidirectional association between Internet usage and emotional disorders. Moderate use of the Internet can serve as an effective source of social support and health information, which helps the older adult maintain social connections and obtain emotional comfort, thereby exerting a protective effect on mental health (12). For instance, a large-scale cross-national study indicates that Internet usage is positively correlated with the improvement of mental health among people aged 50 and above (13). However, this association has a “double-edged sword” effect: excessive or problematic Internet use may lead to sleep deprivation, Internet addiction, and activities that are detached from real social interaction, which in turn exacerbates anxiety and depression symptoms (14, 15). This complexity may be more prominent among rural older adult groups with uneven digital access and limited health literacy. Although relevant research has focused on adolescents and urban older adult, high-quality empirical evidence for rural older adult in China—a group facing both digital and health vulnerabilities—remains extremely scarce. Therefore, clarifying the association pattern and mechanism of action between Internet usage and emotional disorders among rural older adult is of great significance for formulating precise rural public health intervention strategies and promoting healthy aging in the digital age.

To address this research gap, the current study utilizes baseline data from the Northern China Lifestyle Medicine Cohort, involving 9,924 adults aged 65 and above from rural areas, to comprehensively analyze the association between internet use and affective disorders. The findings aim to provide an empirical foundation for developing digital health interventions and mental health support strategies tailored to this vulnerable population.

## 2 Methods

### 2.1 Study design and participants

**Figure 1:**
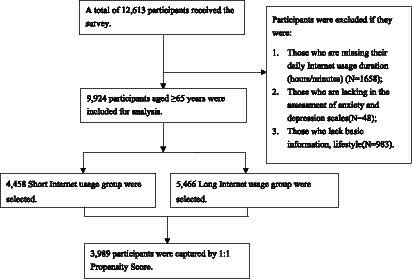
The flowchart of participants selection of this study.

The data were collected from the Northern China Healthy Lifestyle Medicine Cohort (Registration No.: ChiCTR2500096200). The baseline survey was conducted from July 2023 to January 2024 using a three-stage cluster random sampling method. Specifically, Shandong, Henan, Qinghai, and Heilongjiang provinces were selected in the first stage. One county (or county-level city) was then randomly chosen from each province, followed by the random selection of a certain number of townships (or communities) within each county. All villages (or neighborhoods) in the selected townships (communities) were included, and older adults aged 65 and above in the National Basic Public Health Services were recruited as potential participants. Those who met the inclusion criteria and provided informed consent were enrolled in the study. The inclusion criteria were as follows: (1) the older adult included in the national basic public health services; (2) age≥65 years; (3) complete physical examination data in the National Basic Public Health Services database; (4) willingness to participate in the study and provision of informed consent. The exclusion criteria were as follows: (1) missing data on daily Internet usage duration (h/min); (2) missing assessments on anxiety or depression scales; (3) missing basic demographic information; (4) missing lifestyle-related data. A total of 9,924 participants were ultimately included in the study. The participant inclusion and exclusion process is illustrated in Figure 1.

### 2.2 Measures

In this study, the exposure (Internet usage duration), outcomes (affective disorders), and covariates (age, gender, education level, economic status, residential status (urban and rural), marital status, occupation, comorbidities, sleep duration, smoking, drinking, physical activity) of this cohort were evaluated.

#### 2.2.1 Internet use schedule

Internet usage duration was assessed as a categorical variable. This study collected the average daily Internet usage time of the participants through questionnaires. The questionnaire questions clearly target activities that require an Internet connection using smart devices such as smartphones, tablets, and computers, for example: using social applications like WeChat, browsing news websites, watching online videos, online shopping, and searching for health information, etc. In particular, we excluded the time spent passively watching traditional (non-connected) TV programs to focus on active and interactive Internet usage behavior. To standardize the measure, hours were converted to minutes (1 h = 60 min) and summed with the reported minutes to obtain the total daily Internet usage duration. Given the absence of established clinical thresholds for daily internet use duration among rural older adults, we categorized usage based on the sample distribution to explore relative associations. Participants were divided into three groups using tertiles of the baseline distribution: short-term users (≤60 min/day; lower 33%), medium-term users (60–120 min/day; middle 33%), and long-term users (>120 min/day; upper 33%). This data-driven approach ensured balanced group sizes for statistical comparison and allowed the analysis to reflect the intrinsic usage patterns within this specific population. To minimize heterogeneity between users, combine the medium-term usage users and the long-term usage users to form a binary variable: the short-term usage users and the medium-long-term usage users.

#### 2.2.2 Affective disorders

In this study, affective disorders were defined as a total score of PHQ-9 or GAD-7 ≥ 5 (16, 17). Specifically: Positive anxiety symptoms: GAD-7 total score ≥5 points; Positive depressive symptoms: PHQ-9 total score ≥5 points; Positive affective disorder: Meeting any of the above criteria. To test the robustness of this definition and improve clinical specificity, we conducted a sensitivity analysis, raised the threshold to ≥10 (typically indicating moderate to severe symptoms), and analyzed the situations of anxiety (GAD-7 ≥ 10) and depression (PHQ-9 ≥ 10) respectively. All regression models adjusted for the same set of covariates (including demographic characteristics, lifestyle factors, etc.).

#### 2.2.3 Covariates

Covariates were selected based on previous research and included demographic factors (age and sex), socioeconomic indicators (education level and economic status), living arrangements (marital status and residence status), lifestyle factors (smoking, alcohol consumption, sleep duration, and physical activity), occupation, and comorbidities.

### 2.3 Statistical analysis

Continuous variables were expressed as mean ± standard deviation, while categorical variables are described as frequency and percentage. First, a univariate analysis was conducted to compare the prevalence of affective disorders among different Internet usage users. Binary Logistic regression model was constructed to assess factors associated with the prevalence of affective disorders. Results were expressed as adjusted Odds ratio (aOR) and their 95% confidence intervals, while adjusting for confounding variables. To further reduce confounding bias, propensity score matching (PSM) analysis was implemented. Taking Internet usage duration (short vs. medium-long) as the treatment variable, the propensity score was estimated based on covariates, and the 1:1 nearest neighbor matching method (with the clam*p* value set at 0.05) was used to evaluate the balance of covariates between users (17). After matching, the differences in the prevalence of affective disorders between the two users were compared to verify the robustness of the main effect. To assess the robustness of our findings to potential unmeasured confounding, we calculated the *E*-value based on the propensity score matched risk difference and its confidence interval. To examine whether there is a potential nonlinear association (such as a U-shaped relationship) between Internet usage duration and affective disorder, we additionally used Restricted Cubic Splines (RCS) to model Internet usage duration as a continuous variable and calculate the *p*-values of the overall association and the nonlinear association. This analysis also adjusted for all covariates such as gender, age, and educational level.

### 2.4 Ethical considerations

The study protocol was reviewed and approved by the Medical Ethics Committee of Zhengzhou University (Approval number: 2023–318). Prior to participation, written informed consent was obtained directly from each participant. All participants were community-dwelling older adults who were deemed capable of providing consent based on the study’s inclusion criteria. All procedures were conducted in accordance with the ethical standards of the Declaration of Helsinki.

## 3 Results

### 3.1 The distribution characteristics of internet usage and the prevalence of affective disorders among the older adult

A total of 9,924 rural older adult aged 65 and above were included, among whom 43.00% were male (*n* = 4,267) and 57.00% were female (*n* = 5,657). The overall prevalence of affective disorders was 18.54%, with females (21.77, 95%CI: 20.70–22.85) significantly higher than males (14.24, 95%CI: 13.19–15.29) (*p* < 0.001). Internet usage was unevenly distributed: the short-term users accounted for 44.92% (*n* = 4,458), the medium-terms users accounted for 29.79% (*n* = 2,956), and the long-terms users accounted for 25.29% (*n* = 2,510). The prevalence of affective disorders varied significantly among the three users. It was the longest in the short-term users (20.59, 95%CI: 19.40–21.77), 17.05% in the medium-terms users (95%CI: 15.69–18.40), and the shortest in the long-terms users (16.65, 95%CI: 15.19–18.11) (*p* < 0.001). The prevalence of affective disorders decreases with the increase of educational level and family income. The prevalence rate was the longest in the illiterate users (23.38, 95%CI: 21.97–24.80), and the shortest in the long school and above education users (12.51, 95%CI: 9.96–15.07). The prevalence rate in the short-income users (21.35, 95%CI: 20.03–22.67) was significantly higher than that in the middle-income users (17.77%) and the long-income users (15.85%) (*p* < 0.001). The detailed characteristics and prevalence distribution are shown in Table 1.

**Table 1:** Characteristics of research participants related to affective disorder.

Variables	Participants (%)	Affective disorder	
		Prevalence rate (95%CI)	*p* value^a^
All participants	9,924 (100.00)	18.54 (17.78–19.31)	
Internet usage duration			<0.001
Short	4,458 (44.92)	20.59 (19.40–21.77)	
Medium	2,956 (29.79)	17.05 (15.69–18.40)	
Long	2,510 (25.29)	16.65 (15.19–18.11)	
Sex			<0.001
Male	4,267 (43.00)	14.24 (13.19–15.29)	
Female	5,657 (57.00)	21.77 (20.70–22.85)	
Age (year)			0.367
65 ~ 79	8,846 (89.14)	18.66 (17.85–19.47)	
80 and above	1,078 (10.86)	17.53 (15.26–19.80)	
Household registration system			0.001
Urban household registration	656 (6.61)	13.87 (11.22–16.51)	
Rural household registration	9,268 (93.39)	18.87 (18.07–19.66)	
Education level			<0.001
Illiterate	3,446 (34.72)	23.38 (21.97–24.80)	
Primary	3,945 (39.75)	16.32 (15.17–17.47)	
Secondary	1886 (19.00)	16.38 (14.71–18.05)	
Upper secondary and above	647 (6.52)	12.51 (9.96–15.07)	
Marital status			0.099
Married	7,568 (76.26)	18.18 (17.31–19.05)	
Others	2,356 (23.74)	19.69 (18.08–21.30)	
Income group			<0.001
Short	3,700 (37.28)	21.35 (20.03–22.67)	
Medium	3,279 (33.04)	17.77 (16.47–19.08)	
Long	2,945 (29.68)	15.85 (14.53–17.17)	
Occupation			0.075
Farmer	6,881 (69.34)	18.07 (17.16–18.98)	
Non-farmer	3,043 (30.66)	19.58 (18.17–20.99)	
Complications			<0.001
No	5,379 (54.20)	14.27 (13.34–15.21)	
Yes	4,545 (45.80)	23.58 (22.35–24.82)	
Sleeping time			<0.001
Unhealthy	3,496 (35.23)	22.45 (21.07–23.83)	
Healthy	6,428 (64.77)	16.41 (15.50–17.31)	
Smoking			<0.001
Non-smoker	1,552 (15.64)	13.07 (11.40–14.75)	
Smoker	8,372 (84.36)	19.55 (18.70–20.40)	
Drinking			<0.001
Unhealthy	1,589 (16.01)	12.96 (11.31–14.61)	
Healthy	8,335 (83.99)	19.60 (18.75–20.45)	
Physical activity			<0.001
Unhealthy	2,217 (22.34)	22.32 (20.59–24.06)	
Healthy	7,707 (77.66)	17.45 (16.60–18.29)	

a Difference between affective disorder within each variable.

### 3.2 Factors influencing affective disorders in internet-using participants

Based on the analysis, which indicated comparable reductions in the risk of affective disorders for the medium (16%) and long (17%) internet usage users compared to the short usage users (see Supplementary Table S1 for details), and considering that a certain level of internet use may confer a protective effect, the original three-category internet usage variable was recoded into a binary variable. The medium-usage users (*n* = 2,956) and long-usage users (*n* = 2,510) were combined into a single “medium-long usage” users (*n* = 5,466) for comparison with the short-usage users (*n* = 4,458). After adjusting for demographic, socioeconomic, and lifestyle factors, the analysis revealed that within both the medium-long internet usage users [aOR = 1.44, 95%CI (1.21–1.72)] and the short usage users [aOR = 1.39, 95%CI (1.15–1.68)], females were significantly more likely to experience affective disorders than males. For the medium-long internet usage users, a healthy sleep duration was significantly associated with a lower prevalence of affective disorders [aOR = 0.68, 95%CI (0.58–0.79)]. Conversely, within the short internet usage users, unhealthy physical activity levels were associated with a higher risk of affective disorders compared to healthy physical activity [aOR = 0.57, 95%CI (0.48–0.68)]. All reported associations were statistically significant (*p* < 0.05), as detailed in Table 2.

**Table 2:** Influencing factors of internet usage.

Variables	Medium and long usage users				Short-usage users			
	OR	*p*	aOR	*p*	OR	*p*	aOR	*p*
Sex								
Male	1.00 (ref.)		1.00 (ref.)		1.00 (ref.)		1.00 (ref.)	
Female	1.65 (1.42–1.91)	<0.001	1.44 (1.21–1.72)	<0.001	1.63 (1.39–1.91)	<0.001	1.39 (1.15–1.68)	<0.001
Age, year								
65 ~ 79	1.00 (ref.)		1.00 (ref.)		1.00 (ref.)		1.00 (ref.)	
80 and above	0.88 (0.69–1.14)	0.360	0.88 (0.67–1.14)	0.340	0.91 (0.73–1.14)	0.452	0.77 (0.61–0.98)	0.036
Household registration system								
Urban household registration	1.00 (ref.)		1.00 (ref.)		1.00 (ref.)		1.00 (ref.)	
Rural household registration	1.11 (0.81–1.51)	0.536	0.96 (0.68–1.36)	0.852	1.91 (1.38–2.66)	<0.001	1.47 (1.04–2.08)	0.027
Education level								
Illiterate	1.00 (ref.)		1.00 (ref.)		1.00 (ref.)		1.00 (ref.)	
Primary	0.69 (0.59–0.82)	<0.001	0.79 (0.66–0.94)	0.008	0.61 (0.51–0.71)	<0.001	0.77 (0.65–0.92)	0.005
Secondary	0.75 (0.62–0.91)	0.005	0.93 (0.75–1.15)	0.518	0.55 (0.44–0.69)	<0.001	0.71 (0.56–0.91)	0.007
Upper secondary and above	0.47 (0.33–0.65)	<0.001	0.57 (0.40–0.81)	0.002	0.52 (0.36–0.76)	0.001	0.72 (0.48–1.08)	0.121
Marital status								
Married	1.00 (ref.)		1.00 (ref.)		1.00 (ref.)		1.00 (ref.)	
Others	1.03 (0.87–1.21)	0.695	0.92 (0.77–1.09)	0.342	1.19 (1.01–1.41)	0.037	1.05 (0.87–1.26)	0.579
Income group								
Short	1.00 (ref.)		1.00 (ref.)		1.00 (ref.)		1.00 (ref.)	
Medium	0.94 (0.79–1.11)	0.512	1.01 (0.84–1.20)	0.924	0.68 (0.57–0.81)	<0.001	0.86 (0.71–1.03)	0.119
Long	0.77 (0.64–0.92)	0.004	0.85 (0.71–1.02)	0.087	0.65 (0.54–0.79)	<0.001	0.86 (0.70–1.05)	0.144
Occupation								
Farmer	1.00 (ref.)		1.00 (ref.)		1.00 (ref.)		1.00 (ref.)	
Non-farmer	1.08 (0.93–1.26)	0.269	1.09 (0.93–1.29)	0.260	1.11 (0.95–1.31)	0.155	1.07 (0.90–1.26)	0.401
Complications								
No	1.00 (ref.)		1.00 (ref.)		1.00 (ref.)		1.00 (ref.)	
Yes	1.74 (1.51–2.01)	<0.001	1.70 (1.47–1.97)	<0.001	1.96 (1.69–2.27)	<0.001	1.77 (1.52–2.07)	<0.001
Sleeping time								
Unhealthy	1.00 (ref.)		1.00 (ref.)		1.00 (ref.)		1.00 (ref.)	
Healthy	0.66 (0.57–0.76)	<0.001	0.68 (0.58–0.79)	<0.001	0.70 (0.60–0.81)	<0.001	0.70 (0.60–0.81)	0.121
Smoking								
Non-smoker	1.00 (ref.)		1.00 (ref.)		1.00 (ref.)		1.00 (ref.)	
Smoker	1.45 (1.18–1.77)	<0.001	1.07 (0.86–1.34)	0.501	1.79 (1.39–2.31)	<0.001	1.13 (0.85–1.50)	0.369
Drinking								
Unhealthy	1.00 (ref.)		1.00 (ref.)		1.00 (ref.)		1.00 (ref.)	
Healthy	1.53 (1.25–1.86)	<0.001	1.14 (0.91–1.42)	0.253	1.69 (1.30–2.18)	<0.001	1.23 (0.92–1.65)	0.143
Physical activity								
Unhealthy	1.00 (ref.)		1.00 (ref.)		1.00 (ref.)		1.00 (ref.)	
Healthy	0.99 (0.84–1.17)	0.966	1.03 (0.86–1.23)	0.728	0.53 (0.45–0.63)	<0.001	0.57 (0.48–0.68)	<0.001

OR, odds ratio; aOR, adjusted odds ratio. Adjusted the sex, age, household registration system, education level, marital status, income group, occupation, complications, sleeping time, smoking, drinking, physical activity.

### 3.3 Propensity score matching analysis

**Figure 2:**
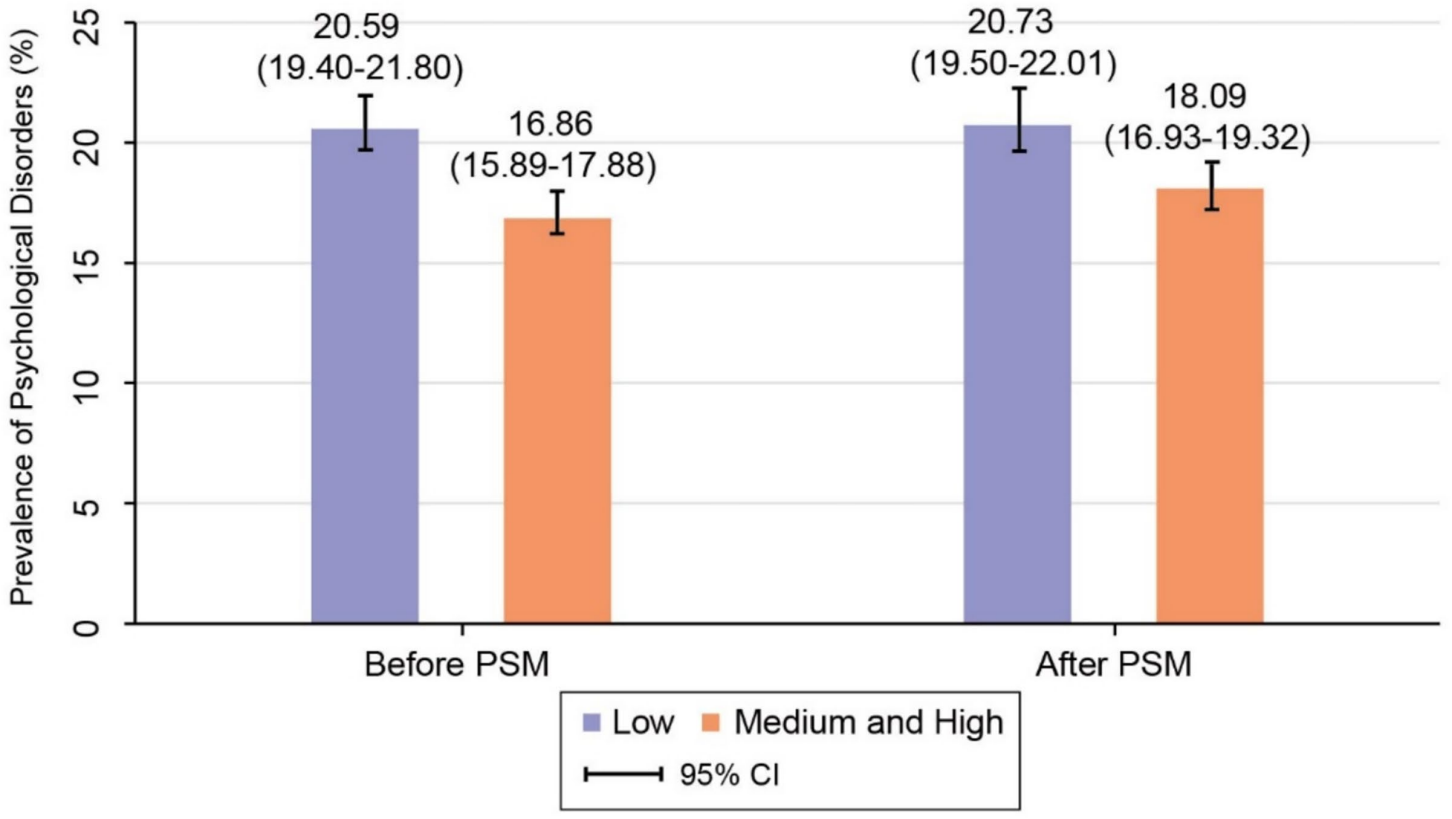
The differences in the prevalence of emotional disorders among short-term internet users and medium- to long-term internet users before and after PSM.

**Figure 3:**
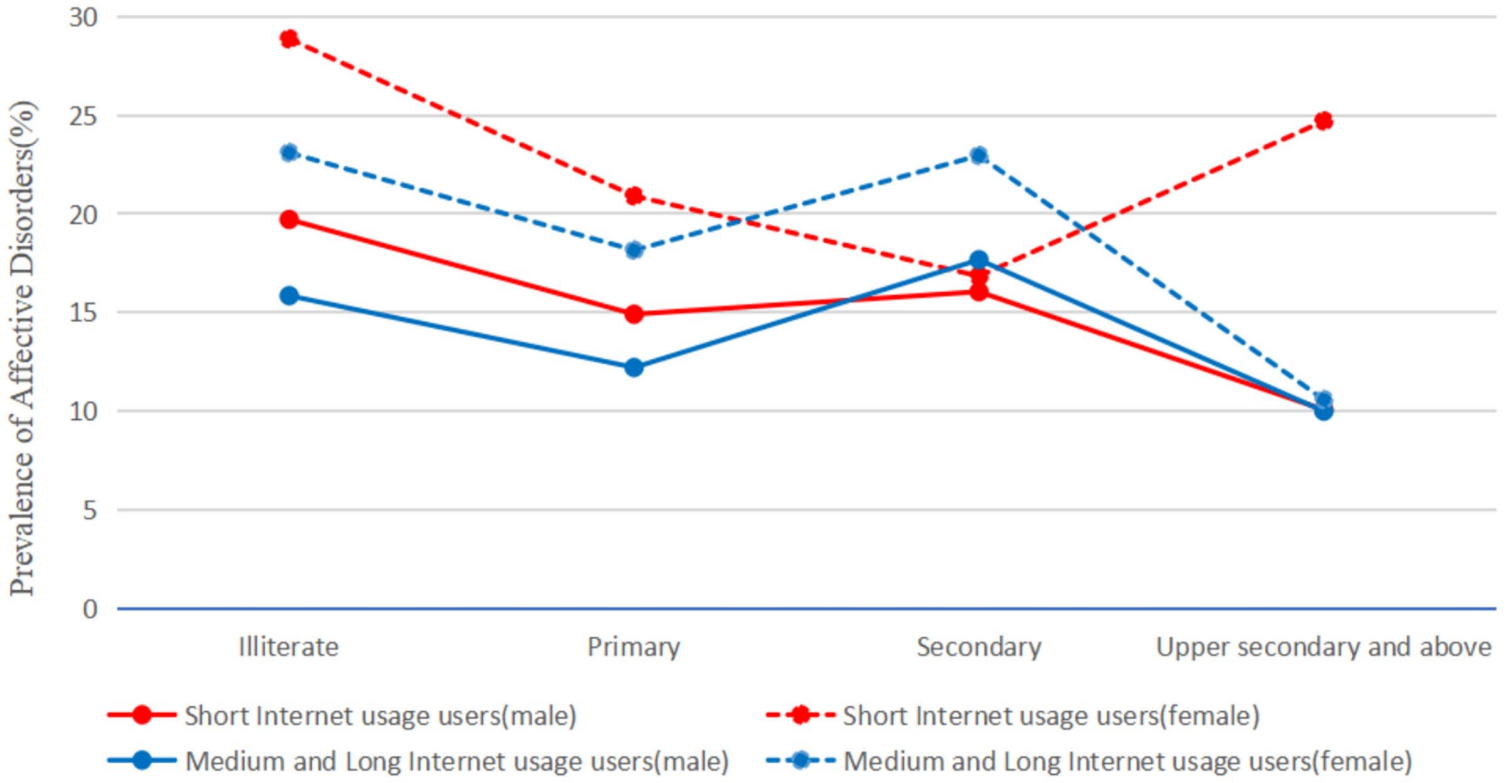
The prevalence of emotional disorders among short-term and medium-long-term internet users after PSM was grouped by educational attainment.

To examine the relationship between Internet use and affective disorders, PSM was applied, producing a matched cohort of 3,989 participants from the total sample of 9,924. After matching, the distributions of key covariates including gender, age, education level, annual household income, marital status, and occupation were balanced between the short internet usage users and the medium-long usage users, with no statistically significant differences observed (all *p* > 0.05). Balance diagnostics and the common support region for the matched samples are provided in Supplementary Table S2 and Supplementary Figure S1, respectively. Based on this balanced sample, the difference in the prevalence of affective disorders between the two users is illustrated in Figure 2. The prevalence remained significantly higher in the short usage users [20.73, 95% CI (19.50–22.01)] compared to the medium-long usage users [18.09, 95% CI (16.93–19.32)], with an absolute difference of 2.64%. Additional supporting details can be found in Supplementary Table S3. Furthermore, when stratified by education level, the prevalence of affective disorders was consistently higher among females than males in both internet usage users. With the exception of females in the short usage users, a general trend of decreasing affective disorder prevalence was observed with higher educational attainment, as shown in Figure 3. To further explore the heterogeneity of the association, we conducted subgroup analyses by gender, age, educational level and comorbidity status (for details, see Supplementary Table S5). The results showed that the association intensity had a changing trend among groups with different educational levels, among which it was most significant among the older adult with illiteracy (OR = 1.31, 95% CI: 1.10–1.54) and primary school education (OR = 1.21, 95% CI: 1.01–1.43). A sensitivity analysis, in which anxiety symptoms and depressive symptoms were examined separately, yielded results consistent with the main analysis regarding the inverse association with internet use duration. Detailed results are presented in Supplementary Table S6.

### 3.4 Dose–response relationship between internet use duration and affective disorders

To explore a more refined association pattern between Internet usage duration and the risk of affective disorders, we conducted a restricted cubic spline analysis (adjusted for all covariates). The results show that there is a significant nonlinear association (nonlinear *p* = 0.001) between Internet usage duration and the risk of affective disorders, and the overall association is also significant (overall *p* < 0.001). As shown in Supplementary Figure S3, the risk of affective disorder generally decreases with the increase of Internet usage duration, especially rapidly within the range of 0–100 min; since then, the curve has flattened out. Even when the usage duration reached 600 min, the risk did not show a U-shaped trend of rebounding above the baseline level. This result further confirms that the protective effect of Internet usage is maintained over a longer usage duration range, supporting the main strategy of combining the analysis of medium and long-term users.

### 3.5 Balance test results

Table 3 reports the results of the covariate balance test before and after propensity score matching. Before matching, there were significant differences between the treatment group and the control group in multiple variables (such as gender, educational level and income group). After matching, the standardized mean bias (%bias) of all variables was significantly reduced, with the vast majority falling below 5%, far below the empirical threshold of 10%. The percentage reduction of deviation after matching generally exceeded 65%, among which the balance improvement of the education level and income groups was particularly obvious. In addition, the *p*-values of the *t*-tests for each variable after matching were all greater than 0.05, and the variance ratios were close to 1. These results indicate that the matching program effectively eliminated the systematic differences in observable features between the treatment group and the control group, meeting the common support hypothesis and balance requirements, and laying a reliable foundation for subsequent causal effect estimation.

**Table 3:** Balance test.

Variable	Unmatched/matched	Mean treated	Mean control	% Bias	Standardized mean difference	% reduct	*t*-test (*p* value)
Sex	U	0.622	0.527	19.3	0.193	9.6	<0.001
	M	0.587	0.595	−1.8	−0.018	90.8	0.426
Age, year	U	1.128	1.093	11.4	0.114	5.7	<0.001
	M	1.097	1.110	−3.9	−0.039	65.6	0.072
Household registration system	U	1.921	1.943	−8.7	−0.087	−4.3	<0.001
	M	1.940	1.935	1.9	0.019	78.1	0.378
Education level	U	1.855	2.069	−24.2	−0.242	−11.9	<0.001
	M	1.928	1.928	0	0	100	1.000
Marital status	U	1.230	1.243	−3.1	−0.031	−1.5	0.125
	M	1.231	1.225	1.6	0.016	48.6	0.472
Income group	U	1.846	1.987	−17.4	−0.174	−8.6	<0.001
	M	1.906	1.896	1.1	0.011	93.4	0.607
Smoking	U	0.872	0.820	14.4	0.144	7.1	<0.001
	M	0.857	0.863	−1.7	−0.017	88.4	0.438
Drinking	U	0.882	0.805	21.4	0.214	10.5	<0.001
	M	0.869	0.861	2.4	0.024	89.0	0.266
Physical activity	U	0.781	0.772	2.0	0.020	1.0	0.311
	M	0.768	0.780	−2.7	−0.027	−32.5	0.228

### 3.6 Exploratory mediation analysis

To examine whether the observed protective association was mediated by improved sleep, we conducted a formal causal mediation analysis. The results indicated that healthy sleep duration did not serve as a significant mediator. The average causal mediation effect (ACME) was negligible and non-significant [ACME = 0.00010, 95% CI (−0.00059–0.00080), *p* = 0.748]. In contrast, the average direct effect of internet use on affective disorders remained significant and negative [ADE = −0.0170, 95% CI (−0.0279 to −0.0064), *p* < 0.001], indicating a protective effect independent of sleep. Detailed results are presented in Supplementary Table S4 and Supplementary Figure S2.

### 3.7 Sensitivity analysis for unmeasured confounding

To quantitatively assess whether unmeasured confounders (e.g., digital literacy, social support) could explain away our primary finding, we calculated the *E*-value. Based on the propensity score matched risk difference [−2.64, 95%CI (−4.38% to −0.91%)], the corresponding risk ratio was 0.87 [95%CI (0.79–0.96)]. The *E*-value was 1.56 for the point estimate and 1.30 for the confidence interval limit closest to the null. This indicates that an unmeasured confounder would need to be associated with both internet use and affective disorders by risk ratios of at least 1.56-fold each to fully explain the observed protective association. Given that the strongest observed confounders in our study (e.g., education, comorbidities) had adjusted odds ratios generally below this threshold, such strong residual confounding seems unlikely.

The calculated *E*-values (1.56 for the point estimate, 1.30 for the confidence interval) provide a quantitative benchmark to assess the potential impact of unmeasured confounding. To contextualize these values, we considered the strength of associations observed for the strongest confounders *already adjusted for* in our study. For instance, the presence of comorbidities—a robust and well-established risk factor for affective disorders—was associated with an adjusted odds ratio (aOR) of approximately 1.7–1.8 in our models. This represents one of the strongest observed associations in our data. The *E*-value suggests that an unmeasured confounder (or a set of confounders) would need to be associated with both internet use and affective disorders by risk ratios exceeding 1.56, above and beyond the adjustment made for powerful covariates like comorbidities, socioeconomic status, and health behaviors. While we cannot rule out residual confounding by factors such as pre-existing subclinical mental states or personality traits (which were not measured), the *E*-value indicates that such confounding would need to be stronger than the strongest known risk factor we accounted for to fully explain away the observed association. This analysis adds a degree of quantitative robustness to our findings against potential unmeasured confounding, though causation cannot be definitively established in this cross-sectional design.

### 3.8 Sensitivity analysis

To improve the clinical specificity of the results, we conducted a sensitivity analysis and defined the threshold of affective disorders as PHQ-9 or GAD-7 ≥ 10. The analysis results show that the protective association of Internet usage duration remains robust. Specifically, compared with short-term use, medium-term and long-term use were significantly associated with a lower risk of anxiety and depression symptoms (for detailed results, see Supplementary Table S7). This result is consistent with the main analysis conclusion, indicating that the research findings are robust to different symptom severity thresholds.

## 4 Discussion

This study investigated the prevalence of affective disorders and its influencing factors among participants categorized into short and medium-long internet usage users. The findings revealed that 18.54% of the study participants were diagnosed with affective disorders. The prevalence rates were 20.59% in the short usage users, 17.05% in the medium usage users, and 16.65% in the long usage users. Healthy sleep duration was shown to be associated with a lower prevalence of affective disorders among participants in the medium-long internet usage users. Within the short usage users, individuals with healthy physical activity levels demonstrated a lower prevalence of affective disorders compared to those with unhealthy physical activity. PSM analysis indicated a 2.64% difference in prevalence between the short and medium-long internet usage users. Furthermore, across all education levels, the prevalence of affective disorders was consistently higher in females than in males, regardless of their internet usage category.

The current study found a higher prevalence of affective disorders among short-term internet users compared to medium and long-term users in a rural older adult cohort, suggesting an inverse association. This observed pattern holds meaningful implications for public health strategies aimed at mitigating the growing burden of affective disorders among China’s aging population, as documented in recent national studies (3). It suggests that promoting appropriate digital engagement could be a novel, scalable component of mental health promotion for rural older adults (18), a subgroup that may benefit substantially from improved social connectivity and access to information and services. This finding is consistent with previous research demonstrating that non-internet users are more susceptible to depressive symptoms, loneliness, and anxiety (7). Moderate internet use can improve psychological status and self-rated health among the older adult, whereas excessive use could have adverse outcomes due to cognitive overload and physiological disruptions (13). Several mechanisms may explain this protective effect. First, internet use enhances communication with family and friends and expands social networks for rural older adult (18, 19), thereby reducing feelings of loneliness and anxiety, which contributes to better mental health (20, 21). Second, using the internet enables older adults in rural areas to access health information, communicate with healthcare professionals, and perform other health-related tasks (19). This facilitates self-health management, such as participation in online health communities (22), leading to improved access to health services and actively promoting their overall health outcomes (23).

The current study identified gender and comorbidities as significant factors strongly associated with the risk of affective disorders among rural older adults. First, after adjusting for multiple confounding variables, females demonstrated a consistently and significantly elevated risk of affective disorders across all levels of internet usage (medium-long usage users: aOR = 1.44; short usage users: aOR = 1.39) (all *p* < 0.05). This finding underscores that older women in rural areas represent a subgroup with particularly long psychological vulnerability. Second, the presence of comorbidities also showed a strong correlation. In both the short and medium-long internet usage users, older adults with chronic conditions exhibited a significantly higher risk of affective disorders compared to those without chronic diseases (medium-long usage users: aOR = 1.70; short usage users: aOR = 1.77) (all *p* < 0.05).

Contrary to a simple health-behavior mediation hypothesis, our formal mediation analysis revealed that the protective association between internet use and lower affective disorder risk was not explained by improvements in sleep duration. This finding suggests that the benefits of digital engagement for rural older adults’ mental health may operate through alternative, more direct psychosocial pathways. These could include reduced loneliness through sustained social connections, enhanced self-efficacy through access to information, or cognitive stimulation from online activities—mechanisms that are not necessarily contingent on improving sleep. This underscores the notion that internet use is a unique behavioral determinant of mental health in late life, with effects that are distinct from, and not merely mediated by, traditional lifestyle factors like sleep. Future research should investigate these direct psychosocial mechanisms, as well as consider whether sleep quality (rather than just duration) or the timing of internet use (e.g., nighttime use) might play a more nuanced moderating role.

Furthermore, when examining the roles of gender and education, we found that within both the short and medium-long internet usage users, the prevalence of affective disorders was consistently higher among women than men across all education levels. Additionally, a trend of decreasing prevalence was observed with higher educational attainment. This aligns with prior research indicating that women generally face a higher risk of affective disorders than men (24). Several factors may explain this disparity. Women in rural areas often have lower educational attainment (25) and disproportionately bear family caregiving responsibilities, which can limit their employment opportunities (26). This may result in poorer internet proficiency and lower health management awareness among older women (27). Moreover, traditional gender norms, such as “men being responsible for outside work and women for domestic duties,” continue to profoundly influence digital engagement and health outcomes among older adults in rural societies. Specifically, older women disproportionately shoulder the responsibility of taking care of their families, whether it be their spouses, grandchildren or older parents. This kind of unpaid labor may lead to social isolation, time deprivation and chronic stress, reducing the opportunities for offline socializing and leisure online activities, which can provide psychological respite. Secondly, due to the higher life expectancy, widowhood is more common among older adult women, which may exacerbate feelings of loneliness and economic insecurity. For those who do not live with children, the lack of immediate social support and potential technological assistance may deepen digital and social exclusion. Therefore, the higher risk of emotional disorders among women is not only a physiological or psychological fact, but also reflects multi-level social vulnerability. Compared to men, older women tend to have lower education levels and limited digital literacy, making them more susceptible to unverified online rumors and pseudo-scientific health advice, thereby missing out on the health benefits offered by the digital era (28). In contrast, men, who often have higher educational attainment, generally possess greater digital and health literacy. This enables them to use the internet more effectively to access medical information, manage their own health, and gain practical smartphone experience through daily use, while also avoiding the negative effects associated with excessive internet use (14, 29). Therefore, policymakers should consider developing targeted internet usage strategies and creating age-friendly digital health platforms tailored to older adults. Efforts should focus on reducing online risks for older women in rural areas and enhancing digital inclusivity (30), thereby shortening the prevalence of affective disorders among this vulnerable demographic.

The subgroup analysis of this study reveals a pattern worthy of in-depth exploration: the intensity of the association between Internet use and affective disorders varies with educational level and shows a non-linear change. The significant positive correlation observed among groups with low educational levels (illiterates, primary school students) suggests that for rural older adult people with the most limited resources, the Internet may play a key compensatory role, being the main or even the only alternative channel for them to obtain information, social support and health services. However, the disappearance of associations in the secondary education group and their resurgence in the high school and above groups jointly paint a complex picture. This might imply that when digital literacy reaches a certain level, the content, quality and context of Internet usage (such as for social comparison, work pressure or high-intensity entertainment) rather than merely the duration, become the dominant factors influencing mental health. Although the statistical heterogeneity of this trend has not reached a significant level (*p* interaction = 0.15), the public health gradient it presents provides important clues for understanding health inequality in the digital age.

The main finding of this study, namely that Internet use is associated with a lower risk of affective disorders, was further verified in the sensitivity analysis. In response to concerns regarding the clinical specificity of diagnostic thresholds, this study adopted stricter clinical criteria (PHQ-9/GAD-7 ≥ 10) and examined anxiety and depression symptoms, respectively, (see Supplementary Table S7 for details). The analysis results show that even with a more clinically significant symptom level as the outcome, the protective association of Internet use (especially medium and long-term use) remains significant and robust. For instance, compared with short-term users, the risk of clinically significant anxiety and depression for medium—and long-term users was significantly reduced (all *p* < 0.01). This finding indicates that the negative association between Internet use and affective disorders may exist under different definitions of symptom severity, thereby enhancing the external validity and clinical reference value of the findings of this study.

### 4.1 Strengths and limitations of the study

This study possesses several distinct strengths. First, it draws on the Northern China Lifestyle Medicine Cohort, which employs a three-stage cluster sampling strategy covering four provinces (Shandong, Henan, Heilongjiang, and Qinghai). By focusing on older adults with chronic conditions managed within rural primary health systems, the study features a large sample size with strong regional representativeness and population specificity. This approach addresses a critical gap in existing research on internet use and mental health, which has predominantly centered on urban or non-chronic disease populations. Second, in terms of variable specification, the study enhances measurement sensitivity and specificity by defining “internet use” through a combination of continuous usage duration and quantile-based using, while assessing affective disorders using both the PHQ-9 and GAD-7 scales. Third, methodologically, the combined application of multivariable logistic regression and PSM establishes an analytical chain ranging from “main effect estimation” to “robustness testing.” This dual approach strengthens the interpretability of the findings and improves the reliability of causal inference, thereby enhancing the translational potential of the results for intervention policy design. Finally, and importantly, this study moves beyond simple association to test a specific mechanistic hypothesis. Our formal mediation analysis allowed us to empirically rule out improved sleep duration as a primary pathway linking internet use to better mental health. This negative finding strengthens the case that the observed protective association operates through more direct psychosocial or digital engagement pathways, thereby refining the theoretical understanding of *how* internet use might benefit rural older adults.

However, this study also has several limitations. First, this cross-sectional study was unable to determine the chronological order of Internet usage and emotional disorders. A reverse causal relationship is a possible explanation. Depression may reduce social activities (including online interactions), or it may increase avoidant Internet use. However, the data show a protective gradient effect: the prevalence of emotional disorders gradually decreases with the increase of daily Internet usage time (short-term: 20.59%, medium-term: 17.05%, long-term: 16.65%). If the reduction in usage (withdrawal hypothesis) is solely due to emotional disorders, it is expected that the prevalence rate among non-users or short-term users will be the highest, but it is difficult to explain the progressive differences that still exist between medium-term and long-term users. Furthermore, propensity score matching balances key confounding factors such as education, income, and comorbidity. Although it cannot rule out reverse causality, it reduces the possibility that the association is entirely driven by these factors. In the future, longitudinal research is needed to track the dynamic relationship between the two to clarify the time sequence. Second, internet usage duration was self-reported, which may be subject to recall bias and under- or over-reporting. Moreover, the lack of granular data on specific online activities (e.g., social networking, entertainment, health information seeking) limits the ability to distinguish between beneficial and harmful usage patterns. Fourth, regarding the generalizability of our findings, it is important to acknowledge the potential for regional bias. Our sample was drawn from four provinces (Shandong, Henan, Heilongjiang, and Qinghai) in Northern China. While this sampling strategy captured a range of socioeconomic and geographic diversity within northern rural China—including major agricultural plains (Shandong, Henan), a northeastern industrial and cold-climate region (Heilongjiang), and a high-altitude western region (Qinghai) with a distinct ethnic composition—it may not fully represent rural older adult populations in other low-resource settings. The socioeconomic development, cultural norms, family structures, and even the nature of digital infrastructure penetration can vary significantly across different regions of China and in other countries. For instance, our findings might be more applicable to rural settings in similar middle-income countries undergoing rapid digital transition, but their direct applicability to rural older adult in high-income countries or in regions with vastly different cultural contexts (e.g., regarding family support or technology adoption) may be limited. Therefore, the conclusions of this study should be interpreted within the context of rural Northern China, and future research is needed to validate these associations in other geographical and cultural settings. Fifth, our exploratory analysis provided clarity on one potential mechanism. We specifically tested and found no evidence that the association was mediated by sleep duration. This actively rules out a common alternative explanation, allowing future research to focus on more promising direct psychosocial mechanisms or on more nuanced factors like sleep quality (rather than duration) as potential moderators. Finally, although multiple covariates were adjusted for in the analysis, potential influential factors such as cognitive function and level of social support were not included. Future research would benefit from incorporating longitudinal data and more comprehensive psychosocial variables to further validate and extend these findings.

## 5 Conclusion

This study found that among the older adult in rural areas, long-term Internet use is associated with a significantly lower prevalence of affective disorders. In terms of policy, efforts should be made to promote the digital integration and mental health advancement of rural older users through infrastructure construction, skills training, and older-friendly health platforms, providing empirical support for achieving digital equity and healthy aging (30).

## Data Availability

The original contributions presented in the study are included in the article/Supplementary material, further inquiries can be directed to the corresponding author.
