# Supplementary material for: Quantifying the association between rural older adult daily internet use duration and affective disorders: an empirical study based on propensity score matching
Source: Front Public Health. 2026 Jan 13;13:1722829. doi: 10.3389/fpubh.2025.1722829 (PMC12834733; doi:10.3389/fpubh.2025.1722829)
Supplement: Supplementary file 1 [file Supplementary_file_1.doc]

**Supplementary Material**

**TableS1.** The association between Internet usage time and psychological disorder

**Table S2.** Balance test of PSM for the low-Internet usage group and the medium and high-Internet usage group.

**Fig. S1.** The common support areas of PSM for both medium and high and low Internet usage groups.

**Table S3** Comparison of the Prevalence of Psychological Disorders Between the Low Internet Use Group and the High Internet Use Group Before and After Propensity Score Matching (PSM).

**Table S4.** Results of Mediation Analysis Testing Sleep Duration as a Mediator of the Association Between Internet Use and Affective Disorders.

**Fig. S2** Schematic representation of the tested mediation model.

**Table S5. Subgroup Analysis of the Association Between Internet Use and Affective Disorders**

**Table S6. Sensitivity analysis: The separate association between Internet usage duration and anxiety and depression**

**Table 7. Sensitivity analysis: Association between internet usage duration and clinically significant anxiety (GAD-7≥10) and depression (PHQ-9≥10) among rural elderly.**

**Figure S3. Restricted cubic spline analysis of the association between daily internet usage dura**tion (minutes) and affective disorders.

| **TableS1.** The association between Internet usage time and psychological disorder | | | | |
| --- | --- | --- | --- | --- |
| **Variables** | Psychological disorder | | | |
|  | OR | *P* | aOR | *P* |
| **Internet use time** |  |  |  |  |
| **Low** | 1.00（ref.) |  | 1.00（ref.) |  |
| **Medium** | 0.79(0.70-0.89) | <0.001 | 0.84(0.75-0.96) | <0.001 |
| **High** | 0.77(0.67-0.87) | <0.001 | 0.83(0.73-0.95) | <0.001 |
| **Sex** |  |  |  |  |
| **Male** | 1.00（ref.) |  | 1.00（ref.) |  |
| **Female** | 1.67（1.50-1.86） | <0.001 | 1.42(1.24-1.61) | <0.001 |
| **Age(year)** |  |  |  |  |
| **65~79** | 1.00（ref.) |  | 1.00（ref.) |  |
| **80 and above** | 0.92（0.78-1.09） | 0.367 | 0.84(0.70-1.01) | 0.056 |
| **Household Registration System** |  |  |  |  |
| **Urban Household Registration** | 1.00（ref.) |  | 1.00（ref.) |  |
| **Rural Household Registration** | 1.44（1.15-1.81） | 0.002 | 1.25(0.98-1.59) | 0.067 |
| **Education level** |  |  |  |  |
| **Illiterate** | 1.00（ref.) |  | 1.00（ref.) |  |
| **Primary** | 0.63（0.56-0.71） | <0.001 | 0.77(0.68-0.87) | <0.001 |
| **Secondary** | 0.64（0.55-0.74） | <0.001 | 0.84(0.72-0.98) | 0.033 |
| **Upper secondary and above** | 0.46（0.36-0.59） | <0.001 | 0.64(0.49-0.83) | 0.001 |
| **Marital status** |  |  |  |  |
| **Married** | 1.00（ref.) |  | 1.00（ref.) |  |
| **Others** | 1.10（0.98-1.24） | 0.099 | 0.98(0.87-1.11) | 0.851 |
| **Income_group** |  |  |  |  |
| **Low** | 1.00（ref.) |  | 1.00（ref.) |  |
| **Medium** | 0.79（0.71-0.89） | <0.001 | 0.93(0.82-1.05) | 0.269 |
| **High** | 0.69（0.61-0.78） | <0.001 | 0.84(0.74-0.97) | 0.018 |
| **Occupation** |  |  |  |  |
| **Farmer** | 1.00（ref.) |  | 1.00（ref.) |  |
| **Non-farmer** | 1.10（0.99-1.23） | 0.075 | 1.09(0.97-1.22) | 0.138 |
| **Comorbidity** |  |  |  |  |
| **No** | 1.00（ref.) |  | 1.00（ref.) |  |
| **Yes** | 1.85（1.67-2.05） | <0.001 | 1.76(1.59-1.96) | <0.001 |
| **Sleeping time** |  |  |  |  |
| **Unhealthy** | 1.00（ref.) |  | 1.00（ref.) |  |
| Healthy | 0.67（0.61-0.75） | <0.001 | 0.70(0.63-0.78) | <0.001 |
| **Smoking** |  |  |  |  |
| Non-smoker | 1.00（ref.) |  | 1.00（ref.) |  |
| Smoker | 1.61（1.38-1.89） | <0.001 | 1.12(0.94-1.33) | 0.195 |
| **Drinking** |  |  |  |  |
| Unhealthy | 1.00（ref.) |  | 1.00（ref.) |  |
| Healthy | 1.63（1.40-1.91） | <0.001 | 1.16(0.97-1.38) | 0.096 |
| **Phy_actitive** |  |  |  |  |
| Unhealthy | 1.00（ref.) |  | 1.00（ref.) |  |
| Healthy | 0.73（0.65-0.82） | <0.001 | 0.77(0.68-0.87) | <0.001 |
| Pseudo R^2^ | 0.0386 | | 0.0387 | |

**Table S2.** Balance test of PSM for the low-Internet usage group and the medium and high-Internet usage group.

| **Variable** | **Pre-PSM** | | | **Post-PSM** | | |
| --- | --- | --- | --- | --- | --- | --- |
|  | **Low Internet usage group** | **Medium and High Internet usage group** | ***P*** | **Low Internet usage group** | **Medium and High Internet usage group** | ***P*** |
| **Sex** | 0.622 | 0.527 | <0.001 | 0.586 | 0.595 | 0.426 |
| **Age(year)** | 1.128 | 1.092 | <0.001 | 1.097 | 1.109 | 0.072 |
| **Household Registration System** | 1.921 | 1.943 | <0.001 | 1.940 | 1.935 | 0.378 |
| **Education level** | 1.855 | 2.069 | <0.001 | 1.928 | 1.928 | 1.000 |
| **Marital status** | 1.230 | 1.243 | 0.125 | 1.231 | 1.225 | 0.472 |
| **Income_group** | 1.846 | 1.987 | <0.001 | 1.906 | 1.896 | 0.067 |
| **Smoking** | 0.872 | 0.820 | <0.001 | 0.857 | 0.863 | 0.438 |
| **Drinking** | 0.882 | 0.805 | <0.001 | 0.869 | 0.860 | 0.266 |
| **Phy_actitive** | 0.781 | 0.772 | 0.311 | 0.768 | 0.780 | 0.228 |


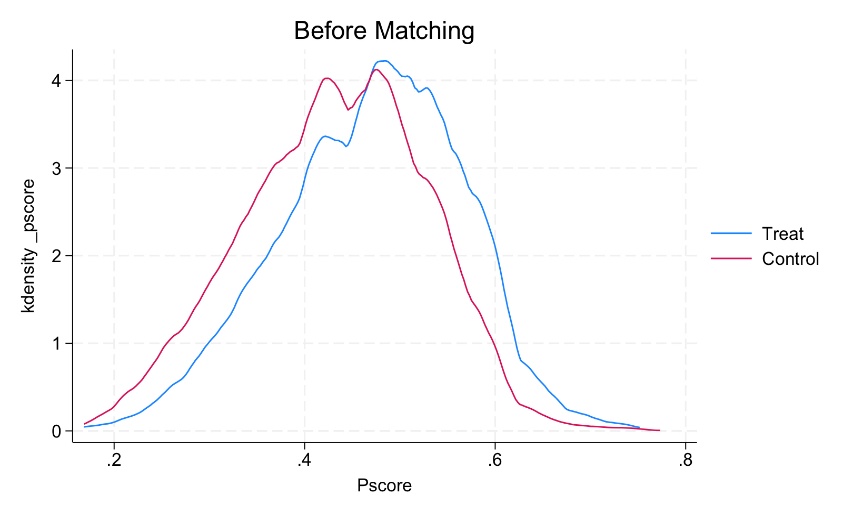

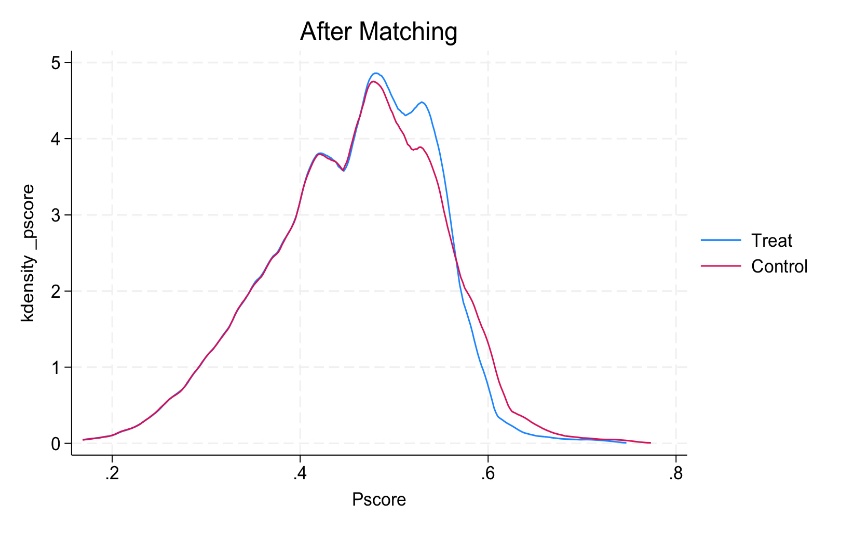


**Fig. S1.** The common support areas of PSM for both medium and high and low Internet usage groups.

| **Table S3** Comparison of the Prevalence of Psychological Disorders Between the Low Internet Use Group and the High Internet Use Group Before and After Propensity Score Matching (PSM). | | | |
| --- | --- | --- | --- |
|  | The Low Internet Use Group | The Medium and High Internet Use Group | *p*^a^ |
| Before PSM | 20.59(19.40-21.80) | 16,86(15.89-17.88) | 0.003 |
| After PSM | 20.73(19.50-22.01) | 18.09(16.93-19.32) | <0.000 |
| a, *p* value of the difference test between the Low Internet Use Group and the Medium and High Internet Use Group | | | |

| **Table S4.** Results of Mediation Analysis Testing Sleep Duration as a Mediator of the Association Between Internet Use and Affective Disorders. | | | | |
| --- | --- | --- | --- | --- |
| Effect | Estimate | 95% CI Lower | 95% CI Upper | P-value |
| Total Effect | -0.0169 | -0.0280 | -0.0065 | <0.001 |
| Average Direct Effect(ADE) | -0.0170 | -0.0279 | -0.0064 | <0.001 |
| Average Causal Mediation Effect(ACME) | 0.0001 | -0.0006 | 0.0008 | 0.748 |
| Proportion Mediated | -0.0062 | -0.0628 | 0.0403 | 0.748 |
| Note: CI = confidence interval. | | | | |

**Fig. S2** Schematic representation of the tested mediation model.

Sleep duration

Internet usage

Affective Disorders

ACME=0.001, *P*=0.784

ADE=-0.017, *P<*0.001

Total Effect=-0.016, *P<*0.001

Prop Mediated=-0.006, *P*=0.784

**Table S5. Subgroup Analysis of the Association Between Internet Use and Affective Disorders**

| Sex | Subgroup | OR | SE | Z | P | 95%CI | Pinteraction |
| --- | --- | --- | --- | --- | --- | --- | --- |
|  |  |  |  |  |  |  | 0.98 |
|  | Male | 1.182 | 0.107 | 1.85 | 0.064 | 0.99-1.41 |  |
|  | Female | 1.171 | 0.078 | 2.37 | 0.018 | 1.02-1.33 |  |
| Age(year) |  |  |  |  |  |  | 0.84 |
|  | 65~79 | 1.175 | 0.066 | 2.85 | 0.004 | 1.05-1.31 |  |
|  | 80 and above | 1.296 | 0.224 | 1.50 | 0.134 | 0.92-1.82 |  |
| Education level |  |  |  |  |  |  | 0.15 |
|  | Illiterate | 1.307 | 0.110 | 3.17 | 0.002 | 1.10-1.54 |  |
|  | Primary | 1.207 | 0.107 | 2.12 | 0.034 | 1.01-1.43 |  |
|  | Secondary | 0.938 | 0.123 | -0.48 | 0.629 | 0.72-1.21 |  |
|  | Upper secondary and above | 1.481 | 0.376 | 1.55 | 0.122 | 0.89-2.43 |  |
| Complications |  |  |  |  |  |  | 0.45 |
|  | No | 1.145 | 0.092 | 1.69 | 0.091 | 0.98-1.34 |  |
|  | Yes | 1.201 | 0.087 | 2.54 | 0.011 | 1.04-1.39 |  |

| **Table 6. Sensitivity analysis: The separate association between Internet usage duration and anxiety and depression** | | | | | | | | |
| --- | --- | --- | --- | --- | --- | --- | --- | --- |
| Variables | Anxiety | | | | Depression | | | |
|  | OR | *P* | aOR | *P* | OR | *P* | aOR | *P* |
| Internet usage duration |  |  |  |  |  |  |  |  |
| Short | 1.00（ref.) |  | 1.00（ref.) |  | 1.00（ref.) |  | 1.00（ref.) |  |
| Medium | 0.52(0.47-0.58) | <0.001 | 0.51(0.45-0.58) | <0.001 | 0.82(0.74-0.91) | <0.001 | 0.86(0.76-0.96) | 0.010 |
| Long | 0.45(0.40-0.50) | <0.001 | 0.44(0.39-0.51) | <0.001 | 0.67(0.61-0.74) | <0.001 | 0.71(0.63-0.81) | <0.001 |
| **Sex** |  |  |  |  |  |  |  |  |
| Male | 1.00（ref.) |  | 1.00（ref.) |  | 1.00（ref.) |  | 1.00（ref.) |  |
| Female | 1.45(1.34-1.55) | <0.001 | 1.49(1.30-1.70) | <0.001 | 1.57（1.46-1.69） | <0.001 | 1.51(1.33-1.71) | <0.001 |
| **Age, year** |  |  |  |  |  |  |  |  |
| 65~79 | 1.00（ref.) |  | 1.00（ref.) |  | 1.00（ref.) |  | 1.00（ref.) |  |
| 80 and above | 0.93(0.83-1.04) | 0.235 | 0.93(0.77-1.13) | 0.474 | 1.03(0.92-1.14) | 0.597 | 1.04(0.87-1.25) | 0.669 |
| **Household Registration System** |  |  |  |  |  |  |  |  |
| Urban Household Registration | 1.00（ref.) |  | 1.00（ref.) |  | 1.00（ref.) |  | 1.00（ref.) |  |
| Rural Household Registration | 0.91(0.79-1.05) | 0.215 | 0.64(0.52-0.78) | <0.001 | 1.39(1.20-1.60) | <0.001 | 1.09(0.90-1.32) | 0.363 |
| **Education level** |  |  |  |  |  |  |  |  |
| Illiterate | 1.00（ref.) |  | 1.00（ref.) |  | 1.00（ref.) |  | 1.00（ref.) |  |
| Primary | 1.15(1.06-1.25) | <0.001 | 1.12(0.99-1.28) | 0.077 | 0.90(0.83-0.98) | 0.018 | 1.00(0.88-1.13) | 0.965 |
| Secondary | 0.84(0.76-0.93) | 0.001 | 0.84(0.71-0.98) | 0.032 | 0.72(0.65-0.79) | <0.001 | 0.84(0.73-0.98) | 0.031 |
| Upper secondary and above | 0.60(0.51-0.71) | <0.001 | 0.75(0.59-0.96) | 0.021 | 0.52(0.45-0.61) | <0.001 | 0.70(0.56-0.88) | 0.002 |
| **Marital status** |  |  |  |  |  |  |  |  |
| Married | 1.00（ref.) |  | 1.00（ref.) |  | 1.00（ref.) |  | 1.00（ref.) |  |
| Others | 0.92(0.85-1.00) | 0.064 | 0.85(0.75-0.97) | 0.019 | 1.16(1.07-1.26) | <0.001 | 1.04(0.92-1.18) | 0.512 |
| **Income group** |  |  |  |  |  |  |  |  |
| Short | 1.00（ref.) |  | 1.00（ref.) |  | 1.00（ref.) |  | 1.00（ref.) |  |
| Medium | 1.37(1.25-1.50) | <0.001 | 1.51(1.32-1.72) | <0.001 | 0.94(0.86-1.03) | 0.194 | 1.08(0.95-1.22) | 0.235 |
| Long | 1.41(1.28-1.54) | <0.001 | 1.01(0.87-1.16) | 0.91 | 1.17(1.07-1.28) | 0.001 | 1.08(0.95-1.24) | 0.249 |
| **Occupation** |  |  |  |  |  |  |  |  |
| Farmer | 1.00（ref.) |  | 1.00（ref.) |  | 1.00（ref.) |  | 1.00（ref.) |  |
| Non-farmer | 0.73(0.68-0.79) | <0.001 | 0.99(0.87-1.12) | 0.851 | 0.98(0.90-1.05) | 0.605 | 1.14(1.01-1.28) | 0.033 |
| **Complications** |  |  |  |  |  |  |  |  |
| No | 1.00（ref.) |  | 1.00（ref.) |  | 1.00（ref.) |  | 1.00（ref.) |  |
| Yes | 1.22(1.14-1.31) | <0.001 | 1.28(1.15-1.42) | <0.001 | 1.37(1.27-1.47) | <0.001 | 1.36(1.23-1.50) | <0.001 |
| **Sleeping time** |  |  |  |  |  |  |  |  |
| Unhealthy | 1.00（ref.) |  | 1.00（ref.) |  | 1.00（ref.) |  | 1.00（ref.) |  |
| Healthy | 0.85(0.79-0.92) | <0.001 | 0.69(0.62-0.77) | <0.001 | 1.06(0.99-1.15) | 0.078 | 0.96(0.86-1.06) | 0.398 |
| **Smoking** |  |  |  |  |  |  |  |  |
| Non-smoker | 1.00（ref.) |  | 1.00（ref.) |  | 1.00（ref.) |  | 1.00（ref.) |  |
| Smoker | 1.07(0.97-1.18) | 0.150 | 0.80(0.69-0.94) | 0.006 | 1.21(1.09-1.33) | <0.001 | 1.06(0.91-1.23) | 0.461 |
| **Drinking** |  |  |  |  |  |  |  |  |
| Unhealthy | 1.00（ref.) |  | 1.00（ref.) |  | 1.00（ref.) |  | 1.00（ref.) |  |
| Healthy | 1.37(1.24-1.52) | <0.001 | 1.07(0.91-1.26) | 0.386 | 1.51(1.36-1.66) | <0.001 | 0.80(0.65-0.98) | 0.033 |
| **Physical activity** |  |  |  |  |  |  |  |  |
| Unhealthy | 1.00（ref.) |  | 1.00（ref.) |  | 1.00（ref.) |  | 1.00（ref.) |  |
| Healthy | 0.76(0.66-0.87) | <0.001 | 0.87(0.70-1.08) | 0.204 | 0.71(0.60-0.81) | <0.001 | 0.97(0.68-1.38) | 0.866 |
| Notes:  OR: Odds ratio; aOR: adjusted Odds ratio. Adjusted the sex, age, household registration system, education level, marital status, income group, occupation, complications, sleeping time, smoking, drinking, physical activity. | | | | | | | | |

| **Table 7. Sensitivity analysis: Association between internet usage duration and clinically significant anxiety (GAD-7≥10) and depression (PHQ-9≥10) among rural elderly.** | | | | | | | | |
| --- | --- | --- | --- | --- | --- | --- | --- | --- |
| Variables | Anxiety | | | | Depression | | | |
|  | OR | *P* | aOR | *P* | OR | *P* | aOR | *P* |
| Internet usage duration |  |  |  |  |  |  |  |  |
| Short | 1.00（ref.) |  | 1.00（ref.) |  | 1.00（ref.) |  | 1.00（ref.) |  |
| Medium | 0.59(0.44-0.79) | 0.001 | 0.67(0.49-0.90) | 0.009 | 0.38(0.26-0.56) | <0.001 | 0.43(0.30-0.64) | <0.001 |
| Long | 0.44(0.31-0.62) | <0.001 | 0.48(0.34-0.69) | <0.001 | 0.54(0.37-0.76) | 0.001 | 0.58(0.40-0.83) | 0.003 |
| **Sex** |  |  |  |  |  |  |  |  |
| Male | 1.00（ref.) |  | 1.00（ref.) |  | 1.00（ref.) |  | 1.00（ref.) |  |
| Female | 1.52(1.17-1.97) | 0.002 | 1.31(0.96-1.81) | 0.092 | 1.51(1.13-2.01) | 0.005 | 1.20(0.85-1.70) | 0.301 |
| **Age, year** |  |  |  |  |  |  |  |  |
| 65~79 | 1.00（ref.) |  | 1.00（ref.) |  | 1.00（ref.) |  | 1.00（ref.) |  |
| 80 and above | 0.94(0.63-1.41) | 0.785 | 0.76(0.50-1.16) | 0.206 | 1.11(0.73-1.70) | 0.606 | 0.78(0.50-1.22) | 0.275 |
| **Household Registration System** |  |  |  |  |  |  |  |  |
| Urban Household Registration | 1.00（ref.) |  | 1.00（ref.) |  | 1.00（ref.) |  | 1.00（ref.) |  |
| Rural Household Registration | 2.27(1.11-4.61) | 0.023 | 1.41(0.68-2.93) | 0.363 | 1.28(0.69-2.36) | 0.429 | 0.94(0.49-1.81) | 0.864 |
| **Education level** |  |  |  |  |  |  |  |  |
| Illiterate | 1.00（ref.) |  | 1.00（ref.) |  | 1.00（ref.) |  | 1.00（ref.) |  |
| Primary | 0.44(0.33-0.59) | <0.001 | 0.56(0.41-0.76) | <0.001 | 0.44(0.31-0.60) | <0.001 | 0.59(0.42-0.83) | 0.002 |
| Secondary | 0.47(0.32-0.68) | <0.001 | 0.65(0.44-0.96) | 0.032 | 0.48(0.32-0.72) | 0.001 | 0.72(0.46-1.11) | 0.131 |
| Upper secondary and above | 0.40(0.21-0.75) | 0.005 | 0.71(0.37-1.36) | 0.300 | 0.51(0.27-0.97) | 0.040 | 0.87(0.45-1.70) | 0.692 |
| **Marital status** |  |  |  |  |  |  |  |  |
| Married | 1.00（ref.) |  | 1.00（ref.) |  | 1.00（ref.) |  | 1.00（ref.) |  |
| Others | 1.00(0.74-1.33) | 0.996 | 0.89(0.65-1.20) | 0.436 | 1.23(0.91-1.68) | 0.169 | 1.08(0.78-1.50) | 0.634 |
| **Income group** |  |  |  |  |  |  |  |  |
| Short | 1.00（ref.) |  | 1.00（ref.) |  | 1.00（ref.) |  | 1.00（ref.) |  |
| Medium | 0.65(0.49-0.87) | 0.003 | 0.84(0.63-1.13) | 0.247 | 0.60(0.43-0.83) | 0.002 | 0.84(0.60-1.18) | 0.313 |
| Long | 0.38(0.27-0.54) | <0.001 | 0.57(0.40-0.82) | 0.002 | 0.46(0.32-0.66) | <0.001 | 0.69(0.47-1.01) | 0.057 |
| **Occupation** |  |  |  |  |  |  |  |  |
| Farmer | 1.00（ref.) |  | 1.00（ref.) |  | 1.00（ref.) |  | 1.00（ref.) |  |
| Non-farmer | 0.71(0.53-0.95) | 0.021 | 0.68(0.50-0.91) | 0.010 | 0.99(0.74-1.34) | 0.990 | 0.90(0.65-1.23) | 0.492 |
| **Complications** |  |  |  |  |  |  |  |  |
| No | 1.00（ref.) |  | 1.00（ref.) |  | 1.00（ref.) |  | 1.00（ref.) |  |
| Yes | 2.42(1.86-3.14) | <0.001 | 2.16(1.66-2.82) | <0.001 | 2.24(1.68-2.98) | <0.001 | 1.97(1.47-2.64) | <0.001 |
| **Sleeping time** |  |  |  |  |  |  |  |  |
| Unhealthy | 1.00（ref.) |  | 1.00（ref.) |  | 1.00（ref.) |  | 1.00（ref.) |  |
| Healthy | 0.71(0.55-0.91) | 0.009 | 0.80(0.62-1.03) | 0.082 | 0.57(0.43-0.75) | <0.001 | 0.63(0.47-0.83) | 0.001 |
| **Smoking** |  |  |  |  |  |  |  |  |
| Non-smoker | 1.00（ref.) |  | 1.00（ref.) |  | 1.00（ref.) |  | 1.00（ref.) |  |
| Smoker | 1.55(1.04-2.31) | 0.028 | 1.02(0.66-1.57) | 0.938 | 1.76(1.11-2.81) | 0.016 | 1.09(0.66-1.80) | 0.750 |
| **Drinking** |  |  |  |  |  |  |  |  |
| Unhealthy | 1.00（ref.) |  | 1.00（ref.) |  | 1.00（ref.) |  | 1.00（ref.) |  |
| Healthy | 1.42(0.97-2.08) | 0.066 | 0.96(0.62-1.47) | 0.840 | 1.92(1.19-3.09) | 0.007 | 1.37(0.81-2.31) | 0.243 |
| **Physical activity** |  |  |  |  |  |  |  |  |
| Unhealthy | 1.00（ref.) |  | 1.00（ref.) |  | 1.00（ref.) |  | 1.00（ref.) |  |
| Healthy | 0.38(0.29-0.49) | <0.001 | 0.41(0.32-0.54) | <0.001 | 0.32(0.24-0.43) | <0.001 | 0.36(0.27-0.49) | <0.001 |
| Notes:  OR: Odds ratio; aOR: adjusted Odds ratio. Adjusted the sex, age, household registration system, education level, marital status, income group, occupation, complications, sleeping time, smoking, drinking, physical activity. | | | | | | | | |

**
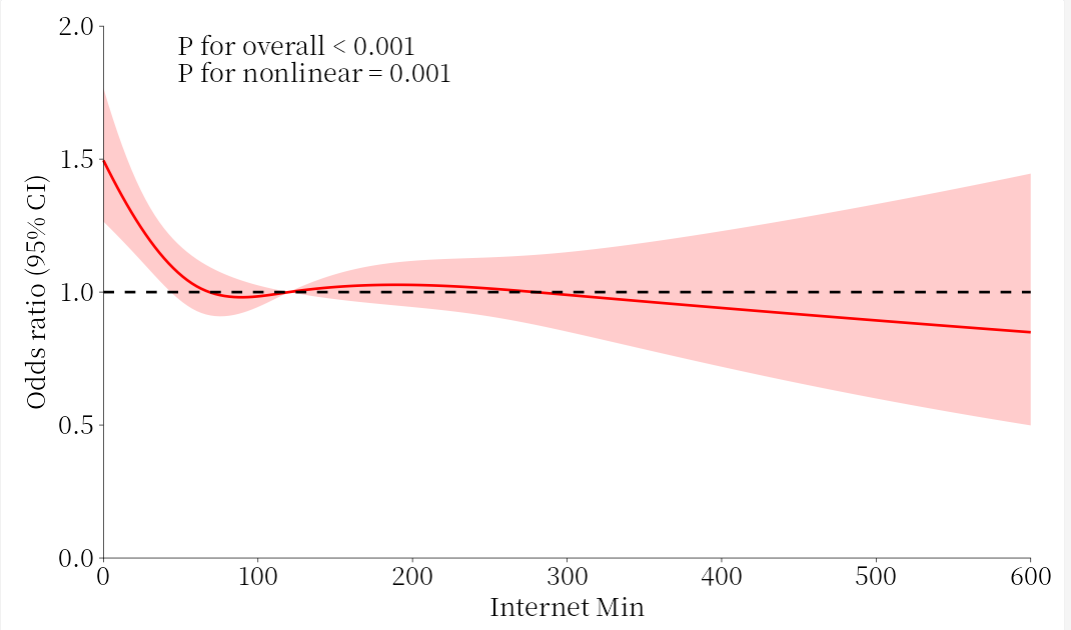
**

**Figure S3. Restricted cubic spline analysis of the association between daily internet usage duration (minutes) and affective disorders.**
